# Summary Measure of Health-Related Quality of Life and Its Related Factors Based on the Chinese Version of the Core Healthy Days Measures: Cross-Sectional Study

**DOI:** 10.2196/52019

**Published:** 2024-07-31

**Authors:** Yulin Shi, Baohua Wang, Jian Zhao, Chunping Wang, Ning Li, Min Chen, Xia Wan

**Affiliations:** 1 Department of Epidemiology and Biostatistics, Institute of Basic Medical Sciences Chinese Academy of Medical Sciences, School of Basic Medicine Peking Union Medical College, 5 Dong Dan San Tiao, Dong Cheng District, Beijing, 100005, China, 8610-65233870; 2 National Center for Chronic and Non-communicable Disease Control and Prevention, Chinese Center for Disease Control and Prevention, Beijing, China; 3 Department of Environmental Hygiene, School of Public Health, Weifang Medical University, Weifang, China; 4 Weifang Center for Disease Control and Prevention, Weifang, China

**Keywords:** health-related quality of life, Healthy Days, summary measure, health status indicators, exploratory factor analyses, confirmatory factor analyses

## Abstract

**Background:**

The core Healthy Days measures were used to track the population-level health status in the China Chronic Disease and Risk Factor Surveillance; however, they were not easily combined to create a summary of the overall health-related quality of life (HRQOL), limiting this indicator’s use.

**Objective:**

This study aims to develop a summary score based on the Chinese version of the core Healthy Days measures (HRQOL-5) and apply it to estimate HRQOL and its determinants in a Chinese population.

**Methods:**

From November 2018 to May 2019, a multistage stratified cluster survey was conducted to examine population health status and behavioral risk factors among the resident population older than 15 years in Weifang City, Shandong Province, China. Both exploratory factor analyses and confirmatory factor analyses were performed to reveal the underlying latent construct of HRQOL-5 and then to quantify the overall HRQOL by calculating its summary score. Tobit regression models were finally carried out to identify the influencing factors of the summary score.

**Results:**

A total of 26,269 participants (male: n=13,571, 51.7%; mean age 55.9, SD 14.9 years) were included in this study. A total of 71% (n=18,663) of respondents reported that they had excellent or very good general health. One summary factor was extracted to capture overall HRQOL using exploratory factor analysis. The confirmatory factor analysis further confirmed this one-factor model (Tucker-Lewis index, comparative fit index, and goodness-of-fit index >0.90; root mean square error of approximation 0.02). Multivariate Tobit regression analysis showed that age (β=–0.06), educational attainments (primary school: β=0.72; junior middle school: β=1.46; senior middle school or more: β=2.58), average income (≥¥30,000 [US $4200]: β=0.69), physical activity (β=0.75), alcohol use (β=0.46), self-reported disease (β=−6.36), and self-reported injury (β=–5.00) were the major influencing factors on the summary score of the HRQOL-5.

**Conclusions:**

This study constructs a summary score from the HRQOL-5, providing a comprehensive representation of population-level HRQOL. Differences in summary scores of different subpopulations may help set priorities for health planning in China to improve population HRQOL.

## Introduction

Health status is a key indicator that reflects the extent of health loss from disease, injury, or other factors affecting physical and mental health [1,2]. In the Global Burden of Diseases study, disability weight was used to quantify the severity of health states from disease sequela and was a pivotal parameter for disease burden calculation [3]. However, it is limited by the complexity of measurement methodology and by the inconsistency between clinical classification description and disease sequela description. For this purpose, series multi-attribute utility instruments based on health-related quality of life (HRQOL) are used to classify health status [1,4]. HRQOL, a multidimensional concept of broad physical and mental health, was used to evaluate overall health and track population health status, health needs, and disparities [5,6]. HRQOL instruments do not assess health status based on diseases or sequelae, but rather on the general population, which is rapidly gaining acceptance as a measurable outcome [7,8].

Based on a synthesis of the scientific literature and advice from its public health partners, the US Centers for Disease Control and Prevention (CDC) defined HRQOL as “an individual’s or group’s perceived physical and mental health over time” and developed a set of Healthy Days measures to track and assess population health status and HRQOL in states and communities [9]. This tool includes four core questions (core Health Days measures [HRQOL-4]) and an additional 10-item set of health perception and activity limitation questions. The CDC HRQOL-4 aims to assess a person’s self-rated general health, poor physical health, poor mental health, and activity limitations. These questions have been well validated in a variety of populations and used fairly extensively in some national surveys in the United States (eg, the Behavioral Risk Factor Surveillance System [BRFSS] and National Health and Nutrition Examination Survey) [9-12]. In addition, the HRQOL-4 has been included in the China Chronic Disease and Risk Factor Surveillance (CCDRFS) since 1996 [13,14]. Given that self-care and usual activities are defined as two different dimensions in some commonly used HRQOL measuring tools (eg, EQ-5D and 36-Item Short Form Survey [SF-36]), the fourth question on activity limitations in the HRQOL-4 was split into two items corresponding to usual activity and self-care limitations, which is called the Chinese version of the core Healthy Days measures (HRQOL-5).

The inclusion of the HRQOL-5 in the CCDRFS has become one of the greatest strengths of the instrument compared to others, as this survey is conducted once every 3 years and provides health-related data from a national sample [15]. No other HRQOL instrument, like the SF-36 or EQ-5D, is accessible in an annual sample as large as that in the CCDRFS. In addition, the government needs to capture poor health statuses with a concise measure due to faster aging and more severe subreplacement fertility in China [16]. The HRQOL-4 differs from other instruments like EQ-5D and SF-36 by not using preference-based measures and not establishing a standardized evaluation system [17]. Previous studies found good internal consistency in the four measures, suggesting that the HRQOL-4 may be suitable for combining a summary index [18]. The US CDC recommended a summary index of “unhealthy days,” using the sum of days of poor physical health (Q2) and days of poor mental health (Q3). However, this index has not been fully validated as a summary of overall health [19]. Some researchers have proposed quantifying the presumed latent HRQOL-4 construct by factor analysis. Horner-Johnson et al [20] and Mielenz et al [21] found that the CDC HRQOL instrument (HRQOL-4 plus the five optional HRQOL module questions) could be reduced to two latent factors that correspond conceptually to the physical and mental health construct. Given that the optional HRQOL module questions were only available for a few national surveys, Yin and colleagues [22] conducted a factor analysis to create a summary score (factor score) by using the CDC HRQOL-4. This summary score showed good validity, stability, and measurement invariance over time in BRFSS data sets, but the feasibility of developing a summary measure based on the HRQOL-5 has not been validated among the Chinese population.

Local levels of population HRQOL may vary from population to population due to differences in socioeconomic status, religion, and lifestyle. Several factors, including age [9,23], sex [9,23], BMI [24], race/ethnicity [9], socioeconomic status [23], tobacco use [25], substance abuse [25], physical activity [26], injury and violence [9], and chronic disease [9,23,25] have previously been studied as correlates of healthy days. However, these studies usually focused only on one or two dimensions of the HRQOL-4 or used univariate analysis that did not take into account the effects of potential confounders. Few studies have evaluated risk factors that may influence population HRQOL based on the summary score of the HRQOL-4.

Therefore, there are two main aims of this study: (1) to propose a summary value based on the HRQOL-5, which could be used to assess overall health status and estimate population-level disability, calculating health-adjusted life expectancy in the future, and (2) to identify factors associated with the summary score of the HRQOL-5 in a Chinese population.

## Methods

### Data Source

Weifang City is located in the area along the eastern coast of Shandong Province in China [27]. In 2021, the total population was 9,372,990, of which 82.6% were 15 years or older. The data used in this study were taken from a cross-sectional survey on population health status and behavioral risk factors among the resident population older than 15 years, carried out by our team from November 2018 to May 2019 in Weifang City [28]. A multistage stratified cluster sampling approach was used to select the sample. Specifically, in the first stage, all township-level administrative regions under Weifang were divided into urban or rural areas as primary sampling units. A total of 29 township-level administrative regions were then selected based on the probability proportionate to size sampling. In the second stage, urban neighborhood committees or rural villages as secondary sampling units were selected from each of the selected primary sampling units using the probability proportionate to size sampling method. The selected secondary sampling unit was partitioned into clusters, with each cluster containing 500 households. Clusters were then selected based on simple random sampling. Finally, one person per household was randomly surveyed. Face-to-face questionnaire surveys were conducted to collect information on participant demographic characteristics, risk factors, self-reported diseases, and HRQOL using tablets or smartphones.

### Ethical Considerations

The proposal for this study was reviewed by the Chinese Academy of Medical Science and School of Basic Medicine (033‐2018). The content and purpose of the study were explained to each participant in advance, and informed consent was obtained from all participants enrolled in this study. Participants could receive a small gift (eg, a towel or facecloth) after completing the survey. Participants were also promised that all the information they provided would be treated confidentially and only used for academic research.

### Statistical Analysis

The HRQOL-5 included 5 questions (Table S1 in Multimedia Appendix 1). The first question (Q1), to measure overall self-rated health, was a 5-level ordinal variable, including excellent, very good, good, fair, and poor. The two subsequent questions (Q2 and Q3) were continuous variables. Questions 4 and 5 (Q4 and Q5) were defined as 4-level ordinal variables (0 days, 1‐6 days, 7‐14 days, and 15‐30 days). Covariates covered 4 broad categories of determinants of health including demographic variables (age, sex, and BMI), socioeconomic variables (educational attainment, marital status, average income, and residence), behavioral variables (smoking, drinking, and physical activity), and health variables (self-reported diseases and self-reported injuries). A BMI below 18.5 kg/m² was categorized as underweight, 18.5‐23.9 kg/m² was considered normal weight, 24.0‐27.9 kg/m² was classified as overweight, and 28.0 kg/m² or higher was designated as obesity [29]. Smoking was defined as consuming at least 1 stick of tobacco [30]. Drinking was defined as consuming alcohol at least once per month over the previous 12 months [31]. Sufficient physical activity was defined as participants meeting any of the following criteria: at least 150 minutes of moderate-intensity physical activity per week, 75 minutes of vigorous-intensity physical activity per week, or any equivalent combination of the two [32]. Participants were asked whether they were diagnosed with any diseases or injuries by a physician. Table 1 shows the assigned values of each covariable.

**Table 1.** The assignments of each covariable.

Variables	Assignments
Age (years)	Continuous variable
Sex	Male=0; female=1
BMI (kg/m^2^)^a^	18.5‐23.9 (normal weight)=0; <18.5 (underweight)=1; 24.0‐27.9 (overweight)=2; ≥28.0 (obesity)=3
Education^a^	Illiterate=0; primary school=1; junior middle school=2; senior middle school or more=3
Marital status^a^	Married=0; unmarried=1; divorced/widowed=2
Average income (¥)^a^^,^^b^	Less than 5000=0; 5000‐9999=1; 10,000‐29,999=2; 30,000 or more=3
Residence	Rural=0; urban =1
Physical activity	No=0; yes=1
Smoking	No=0; yes=1
Drinking	No=0; yes=1
Self-reported disease	No=0; yes=1
Self-reported injury	No=0; yes=1

a Entered into the regression model as the dummy variable.

b A currency exchange rate of ¥1=US $0.14 is applicable.

Exploratory factor analysis (EFA) and confirmatory factor analysis (CFA) with 5 HRQOL items were conducted to reveal the underlying latent construct of the HRQOL-5. Given that a factor structure derived from EFA will almost always fit in CFA on the same sample, the data in this study was randomly split into two samples, including a derivation sample (sample 1) and a validation sample (sample 2) [33]. Standardized Cronbach α was calculated to evaluate the internal consistency or reliability due to the large variance difference among the five items, with a cutoff value of 0.70 or higher considered statistically acceptable [34]. Similar to other studies, Cronbach α when the item was removed was used to test the reliability of the HRQOL-5 further [20,22]. Before performing EFA, the Kaiser-Meyer-Olkin statistic and Bartlett test for sphericity were used to assess whether the data was suitable for conducting the factor analysis. The principal axis factoring with the rotation of orthogonal varimax rotation was chosen to test the loading strength of items on factors, which can accommodate variables for nonnormal distribution [22]. The factors with an eigenvalue ≥1 were considered acceptable, and items were assigned to a factor if their factor loadings were equal to or higher than the minimal acceptable cutoff value of ±0.3 [22].

The factor construct from EFA was then further identified by using CFA. The asymptotically distribution-free method was used to account for the nonnormality of variables [35]. Five common goodness-of-fit indicators were used to assess model fit, including Tucker-Lewis index (TLI), comparative fit index (CFI), goodness-of-fit index (GFI), standardized root mean square residual (SRMR), and the root mean square error of approximation (RMSEA). The traditional cutoff values regarded as a good fit for models are as follows: TLI ≥0.90, CFI ≥0.90, GFI ≥0.90, SRMR ≤0.08, and RMSEA ≤0.08 [33]. We calculated the summary score (factor score) of the HRQOL-5 by the final CFA model. This summary score could be identified as weighted sum scores (multiplying the standard [*z*] scores of each item into its factor loading and then summing them), with a lower value indicating worse HRQOL. For ease of understanding, the minimum factor score was anchored to 0, which meant full health losses (Q1: poor; Q2: 30 days; Q3: 30 days; Q4: 15‐30 days; Q5: 15‐30 days). The maximum score was anchored to 100, which was defined as full health (Q1: excellent; Q2: 0 days; Q3: 0 days; Q4: 0 days; Q5: 0 days), and the rest of the scores were transformed into a 0‐100 range using min-max normalization.

Frequencies and percentages were calculated for categorical variables, and the mean and SD were calculated for quantitative variables. Given the ceiling effect of the HRQOL-5, a Tobit regression model (also known as the censored model) was chosen to identify statistically significant variables affecting the summary score of the HRQOL-5 [19]. The severity of multicollinearity in Tobit regression was assessed by the variance inflation factor with a cutoff value of <10 [33].

In this study, all *P* values <.05 were considered statistically significant. The 95% CIs were estimated for the regression coefficients. All the data analysis was conducted using SPSS Statistics 26.0 (IBM Corp), Amos 24.0 software, and R V.4.1.0 (R Foundation for Statistical Computing). All methods were performed in accordance with the relevant guidelines and regulations.

## Results

### Characteristics of Participants

Table 2 shows the demographic characteristics and measures of HRQOL of the 26,269 participants in Weifang, China (male: n=13,573, 51.7%; from urban areas: n=13,685, 52.1%). The average age was 55.9 (range from 15 to 98) years. Less than 20% (n=5040) of respondents had a senior middle school or high education. For the core Healthy Days measures, none of the measures demonstrated floor effects; however, four measures (Q2-Q5) showed ceiling effects (ie, 24,364, 92.8% reported 0 days of poor physical health; n=24,989, 95.1% reported 0 days of poor mental health, and over 97% reported 0 days of usual activity limitation or self-care limitation). The skewed distributions also were obvious in Q2 and Q3 from the HRQOL-5 result for a median of 0 (IQR 0-0) days, with a mean of 1.0 (SD 4.8) and 0.5 (SD 3.1) days, respectively. Overall, approximately 70% (n=18,663) of participants reported having excellent (n=5773, 21.9%) or very good (n=12,890, 49.1%) health. In addition, more than 90% (n=24,022) of participants responded with “full healthy days,” including 0 days of poor physical or mental health or limitations in usual activity or self-care (Table 2). The final sample size of the derivation sample (sample 1) was 13,078, and the confirmatory sample (sample 2) was 13,191. The results also show that there was no statistical difference between the two samples across all variables (Table 2).

**Table 2.** Social demographic characteristics and health-related quality of life measures of the 26,269 participants in Weifang, China (2018-2019).

Variable				Total sample (N=26,269)		Sample 1 (n=13,078)		Sample 2 (n=13,191)		Chi-square (*df*)	*P* value
**Basic information**											
	**Sex, n (%)**									0.29 (1)	.60
		Male		13,573 (51.7)		6779 (51.8)		6794 (51.5)			
		Female		12,696 (48.3)		6299 (48.2)		6397 (48.5)			
	Age (years), mean (SD)			55.9 (14.9)		56.0 (14.8)		55.8 (14.9)		0.90 (1)	.37
	**BMI, n (%)**									0.64 (3)	.42
		Normal weight		11,498 (43.8)		5674 (43.4)		5824 (44.2)			
		Underweight		1445 (5.5)		723 (5.5)		722 (5.5)			
		Overweight		9783 (37.2)		4940 (37.8)		4843 (36.7)			
		Obesity		3543 (13.5)		1741 (13.3)		1802 (13.7)			
	**Education, n (%)**									0.14 (3)	.71
		Illiterate		4156 (15.8)		2094 (16.0)		2062 (15.6)			
		Primary school		7192 (27.4)		3512 (26.9)		3680 (27.9)			
		Junior middle school		9881 (37.6)		4952 (37.9)		4929 (37.4)			
		Senior middle school or more		5040 (19.2)		2520 (19.3)		2520 (19.1)			
	**Marital status, n (%)**									0.01 (2)	.93
		Married		22,934 (87.3)		11,430 (87.4)		11,504 (87.2)			
		Unmarried		840 (3.2)		389 (3.0)		451 (3.4)			
		Divorced/widowed		2495 (9.5)		1259 (9.6)		1236 (9.4)			
	**Average income (¥)^a^, n (%)**									1.89 (3)	.17
		<5000		10,378 (39.5)		5189 (39.7)		5189 (39.3)			
		5000‐9999		4721 (18.0)		2374 (18.2)		2347 (17.8)			
		10,000‐29,999		6664 (25.4)		3281 (25.1)		3383 (25.7)			
		30,000 or more		4506 (17.2)		2234 (17.1)		2272 (17.2)			
	**Residence, n (%)**									0.29 (1)	.59
		Rural		12,584 (47.9)		6243 (47.7)		6341 (48.1)			
		Urban		13,685 (52.1)		6835 (52.3)		6850 (51.9)			
	**Physical activity, n (%)**									1.74 (1)	.19
		No		3813 (14.5)		1936 (14.8)		1877 (14.2)			
		Yes		22,456 (85.5)		11,142 (85.2)		11,314 (85.8)			
	**Smoking, n (%)**									0.02 (1)	.88
		No		20,617 (78.5)		10,259 (78.4)		10,358 (78.5)			
		Yes		5652 (21.5)		2819 (21.6)		2833 (21.5)			
	**Drinking, n (%)**									2.03 (1)	.15
		No		19,834 (75.5)		9924 (75.9)		9910 (75.1)			
		Yes		6435 (24.5)		3154 (24.1)		3281 (24.9)			
	**Self-reported disease, n (%)**									0.07 (1)	.79
		No		18,319 (69.7)		9130 (69.8)		9189 (69.7)			
		Yes		7950 (30.3)		3948 (30.2)		4002 (30.3)			
	**Self-reported injury, n (%)**									0.92 (1)	.34
		No		25,661 (97.7)		12,787 (97.8)		12,874 (97.6)			
		Yes		608 (2.3)		291 (2.2)		317 (2.4)			
**Health-related quality of life**											
	**General health status, n (%)**									0.65 (4)	.42
		Excellent		5773 (21.9)		2825 (21.6)		2948 (22.4)			
		Very good		12,890 (49.1)		6440 (49.2)		6450 (48.9)			
		Good		5480 (20.9)		2768 (21.2)		2712 (20.6)			
		Fair		2001 (7.6)		988 (7.6)		1013 (7.7)			
		Poor		125 (0.5)		57 (0.4)		68 (0.5)			
	**Days of poor physical health**									0.17 (1)	.68
		Mean (SD)		1.0 (4.8)		1.1 (4.9)		1.0 (4.7)			
		Median (IQR)		0 (0-0)		0 (0-0)		0 (0-0)			
	**Days of poor mental health**									0.14 (1)	.75
		Mean (SD)		0.5 (3.1)		0.5 (3.1)		0.5 (3.1)			
		Median (IQR)		0 (0-0)		0 (0-0)		0 (0-0)			
	**Days of usual activities limitation, n (%)**									2.07 (3)	.15
		0		25,583 (97.4)		12,711 (97.2)		12,872 (97.6)			
		1‐6		112 (0.8)		126 (1.0)		97 (0.7)			
		7‐14		104 (0.4)		54 (0.4)		50 (0.4)			
		≥15		359 (1.4)		187 (1.4)		172 (1.3)			
	**Days of self-care limitation, n (%)**									4.38 (3)	.04
		0		25,887 (98.6)		12,868 (98.4)		13,019 (98.7)			
		1‐6		132 (0.5)		70 (0.5)		62 (0.5)			
		7‐14		63 (0.2)		35 (0.3)		28 (0.2)			
		≥15		187 (0.7)		105 (0.8)		82 (0.6)			

a A currency exchange rate of ¥1=US $0.14 is applicable.

### Factor Analysis

#### Reliability: Internal Consistency

The internal consistency for the HRQOL-5 on the validation sample (n=13,078), measured by Cronbach α, was 0.75, which was within the acceptable range (>0.70). The α change caused by the item removal test indicated good consistency within items. Cronbach α based on standardized items was lowered when other items were removed except for the general health status item. In addition, the removal of items on “days of mental health,” “days of usual activity limitation,” or “days of self-care limitation” reduced the α to <0.70 (Table 3).

**Table 3.** Consistency of the Chinese version of the core Healthy Days measures (HRQOL-5).

Item	Cronbach α^a^	Change (%)
Overall construct	0.75	—^b^
General health status	0.77	+0.02
Days of poor physical health	0.73	–0.02
Days of poor mental health	0.67	–0.08
Days of usual activity limitation	0.64	–0.11
Days of self-care limitation	0.69	–0.06

a Based on standardized items if item was removed.

b Not applicable.

#### Factor Structure: EFA

The data in sample 1 satisfied the requirements for carrying out the factor analysis because the Kaiser-Meyer-Olkin value (0.73) was greater than the cutoff value (0.70), and the Bartlett test of sphericity was also statistically significant (*P*<.001). For the HRQOL-5, a single-factor model (an eigenvalue >1) was proposed by EFA, which accounted for 51% of the total variance (Table S2 in Multimedia Appendix 1).

#### Factor Model: CFA

**Figure 1.**
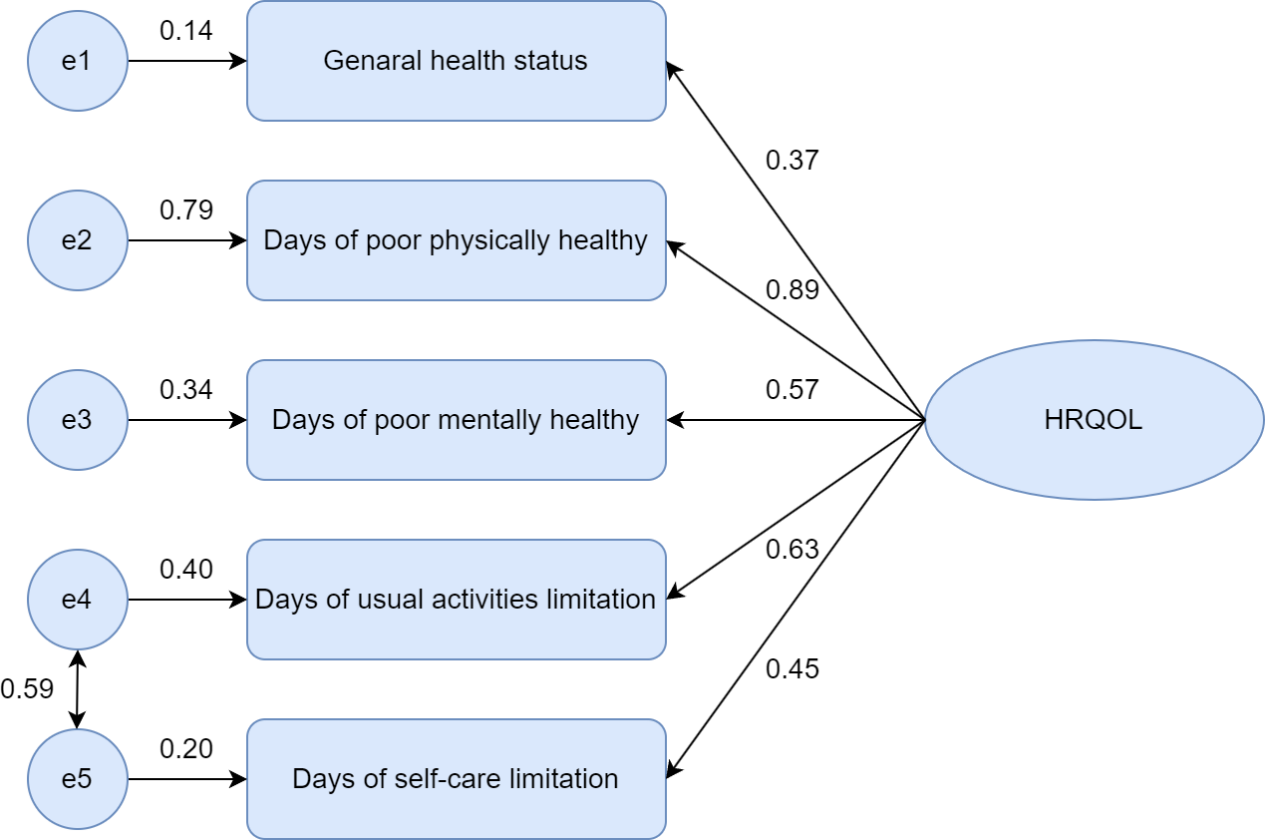
Confirmatory factor analysis for the Chinese version of the core Healthy Days measures (HRQOL-5). HRQOL: health-related quality of life.

We fit an initial model on the validation sample (n=13,191) based on the result of the CFA, which included 5 paths from 1 factor and an error correlation path between the usual activity limitation days item and the self-care limitation days item. This model was acceptable for the data since the goodness-of-fit indicators (TLI 0.93, CFI 0.97, GFI 0.99) were above 0.90, and the RMSEA (0.02) and SRMR (0.01) values were below 0.08. Figure 1 shows a final model for the HRQOL-5; five items had factor loadings that ranged from 0.37 to 0.89 and were significant, which indicated a good relationship between the latent variable and observed variable.

#### Factors Associated With the Summary Score of the HRQOL-5

Univariate analysis indicated that the summary score of the HRQOL-5 was different among groups by sex, BMI, educational attainment, marital status, average income, residence, smoking, drinking, physical activity, self-reported diseases, and self-reported injuries (all *P*<.05; Table 4). Variance inflation factors for all covariates in the Tobit model were <2, suggesting that multicollinearity was not a substantive concern in our study (Table S3 in Multimedia Appendix 1). Thus, no variable was excluded from further multivariable analysis. Table 4 presents the results of the estimation of the multivariate Tobit model. Seven variables (age, educational attainment, average income, drinking, physical activity, self-reported diseases, and self-reported injuries) were significantly associated with scores in the final model. A higher summary score of HRQOL was consistently associated with higher educational attainments (primary school: β=0.72; junior middle school: β=1.46; senior middle school or more: β=2.58), average income (≥¥30,000 [US $4200]: β=0.69), and physical activity (β=0.75). In addition, those who drank alcohol (β=0.46) had higher summary scores. The summary score of the HRQOL-5 was negatively associated with age (β=–0.06), self-reported disease (β=–6.36), and self-reported injury (β=–5.00).

**Table 4.** Univariate and multivariate Tobit regression analysis of factors associated with the summary score on the Chinese version of the core Healthy Days measures (HRQOL-5) in Weifang, China (2018‐2019)

Variables		Summary score, mean (SD)	Univariate		Multivariate	
			β (95% CI)	*P* value	β (95% CI)	*P* value
Age		95.88 (9.41)	–0.17 (–0.18 to –0.16)	<.001	–0.06 (–0.08 to –0.05)	<.001
**Sex**						
	Male	96.16 (9.23)	Reference	—^a^	Reference	—
	Female	95.57 (9.78)	–0.88 (–1.16 to –0.60)	<.001	0.20 (–0.13 to 0.53)	.24
**BMI**						
	Normal weight	95.94 (9.58)	Reference	—	Reference	—
	Underweight	93.99 (12.82)	–2.43 (–3.05 to –1.81)	<.001	–0.52 (–1.12 to 0.08)	.09
	Overweight	96.06 (8.78)	0.03 (–0.28 to 0.34)	.85	0.30 (0.00 to 0.59)	.05
	Obesity	95.93 (8.80)	–0.11 (–0.54 to 0.32)	.61	0.36 (–0.05 to 0.77)	.09
**Education**						
	Illiterate	93.42 (13.02)	Reference	—	Reference	—
	Primary school	94.80 (11.01)	1.66 (1.24 to 2.09)	<.001	0.72 (0.29 to 1.14)	.001
	Junior middle school	96.73 (7.54)	4.47 (4.06 to 4.87)	<.001	1.46 (1.00 to 1.92)	<.001
	Senior middle school or more	97.76 (5.11)	6.51 (6.05 to 6.98)	<.001	2.58 (2.03 to 3.12)	<.001
**Marital status**						
	Married	96.05 (9.02)	Reference	—	Reference	—
	Unmarried	96.63 (10.08)	2.44 (1.61 to 3.26)	<.001	–0.24 (–1.06 to 0.59)	.58
	Divorced/widowed	93.99 (12.14)	–2.95 (–3.42 to –2.49)	<.001	0.26 (–0.21 to 0.73)	.28
**Average income (¥)^b^**						
	<5000	93.57 (9.12)	Reference	—	Reference	—
	5000‐9999	95.47 (8.26)	–0.11 (–0.56 to 0.35)	.64	–0.01 (–0.45 to 0.43)	.96
	10,000‐29,999	96.19 (6.45)	0.91 (0.53 to 1.30)	<.001	0.32 (–0.05 to 0.69)	.09
	≥30,000	97.05 (4.57)	2.41 (2.07 to 2.76)	<.001	0.69 (0.35 to 1.03)	<.001
**Residence**						
	Rural	96.34 (8.66)	Reference	—	Reference	—
	Urban	95.45 (10.03)	–1.39 (–1.67 to –1.11)	<.001	–0.21 (–0.48 to 0.07)	.14
**Physical activity**						
	No	94.50 (11.83)	Reference	—	Reference	—
	Yes	96.11 (8.92)	2.47 (2.08 to 2.86)	<.001	0.75 (0.37 to 1.13)	<.001
**Smoking**						
	No	95.73 (9.74)	Reference	—	Reference	—
	Yes	96.41 (8.08)	0.90 (0.56 to 1.24)	<.001	0.18 (–0.19 to 0.56)	.34
**Drinking**						
	No	95.66 (9,94)	Reference	—	Reference	—
	Yes	96.56 (7.50)	1.20 (0.88 to 1.53)	<.001	0.46 (0.08 to 0.81)	.02
**Self-reported disease**						
	No	97.60 (5.12)	Reference	—	Reference	—
	Yes	91.90 (14.48)	–7.66 (–7.95 to –7.38)	<.001	–6.36 (–6.66 to –6.06)	<.001
**Self-reported injury**						
	No	96.01 (9.10)	Reference	—	Reference	—
	Yes	90.15 (17.34)	–6.91 (–7.81 to –6.01)	<.001	–5.00 (–5.85 to –4.15)	<.001

a Not applicable.

b A currency exchange rate of ¥1=US $0.14 is applicable.

## Discussion

To our knowledge, this is the first study to test the underlying latent construct of the core Healthy Days measures and factors associated with HRQOL in major cities of mainland China. The primary aim of the study extends the results by first using factor analysis to obtain a modified one-factor structure and a summary score based solely on the HRQOL-5. The second aim was to identify possible predictors of the summary score of the HRQOL-5 in a Chinese population. More specifically, age, education, average income, drinking, physical activity, and self-reported disease or injury were significantly associated with the summary score of the HRQOL-5.

The HRQOL-5 distinguished between usual activities and self-care activities in activity limitations and added only one question compared to the HRQOL-4 [14]. The use of the HRQOL-5 might be one of the most cost-effective ways of tracking general health needs in China. The results of the reliability analysis also indicated that the HRQOL-5 had acceptable internal consistency (Cronbach α >0.70). One subtle difference is that in our sample, the Cronbach α based on standardized items increased if the first question on general health status was removed as in previous research [22]. However, because of a small increase (+0.02) in our study and the lack of a clear cutoff value for the increase of the α value, we cannot suggest the internal consistency between the general health status item and the four other questions.

Credible construct validity of the HRQOL-5 was established by conducting both exploratory and CFA for our sample, as evidenced by a series of model goodness-of-fit indicators (RMSEA 0.02, TLI 0.93, GFI 0.99, SRMR 0.01). Previous studies, which used data obtained from the HRQOL-4 questions on the BRFSS, developed and tested a one-factor model [22]. Our results showed similar constructs of HRQOL and item factor loadings. As expected from the CDC HRQOL-4, one distinctive dimension that encompasses both physical and mental health was extracted, thus making it possible to compare the CDC HRQOL-4 and the HRQOL-5. It was noteworthy that a positive error correlation path between the days of usual activity limitation item and the days of self-care limitation item was found. There are at least two theoretical reasons not based on statistical support. First, the format of the fourth question on usual activity limitation and the fifth question on self-care limitation is very similar. Some research has found that using a similar question format may contribute to the covariance between two items, which can affect survey responses [22]. Second, Clifford [36] suggested the possibility of correlated error whenever survey questions are connected physically. Our model may account for this relation by indicating a positive correlation path between the error terms in the measures of usual activity limitation and self-care limitation. Overall, the HRQOL-5 was found to be largely similar to the CDC HRQOL-4, which showed that the underlying structure of HRQOL is consistent across different cultures and languages, thereby lending support to a claim that summary scores of the HRQOL-5 would be simpler and more comprehensive than using the single Healthy Days question [20,22].

Understanding the associations between different HRQOL instruments is becoming a higher priority for government agencies so that they can interpret the potential benefits of different health policies from studies that use either of these instruments. A systematic review shows that the EQ-5D and SF-36 were the most commonly used measure instruments for assessing quality of life [1]. Jia and colleagues [37] developed a mapping algorithm linking the HRQOL-4 and the EQ-5D, and this mapping score might provide a good measurement of individuals’ overall health status [38]. A national study from the United States explored associations between the HRQOL-4 and the SF-36 [39]. In addition, these studies also show differences in health status between the HRQOL-4 and other instruments due to their respective descriptive systems and assessment methods. Thus, there is debate over which instrument can better assess the overall HRQOL in a population. From the cost and efficiency perspective, Healthy Days seems to be more advantageous, since the HRQOL-4 or HRQOL-5 has been included in the national surveillance system in China or the United States. Thus, the national factor scores can be calculated without extra surveys. Additionally, we evaluated the overall health status by considering the potential construct in 5 HRQOL-5 items. It does not need to map to other generic instruments. The summary score based on the HRQOL-5 can be directly applied to various scenarios in China, such as health status evaluation, risk factor identification, and burden of disease assessment in future studies.

The analysis of associations between the summary scores of the HRQOL-5 with the other collected variables potentially affecting HRQOL among the Chinese population has allowed us to confirm data already available in the literature. Consistent with previous studies, age was a significant predictor summary score of the HRQOL-5 [21,40,41]. The health problems became increasingly serious with age, resulting in lower summary scores. Socioeconomic status appeared to be associated with HRQOL as indicated in this study and others [40,41]. Participants who were wealthier and had a higher level of education had higher HRQOL-5 summary scores. In addition, our results showed a positive association between physical activity and the HRQOL level. A national survey in the United States also found a dose-response relationship between unhealthy days and physical activity level [26]. Participants who reported diseases had worse HRQOL than those without. The summary scores of the HRQOL-5 were substantially decreased by self-reported diseases. Substantial evidence showed that self-reported diseases had been considered the main risk factor impairing HRQOL [42,43]. Another interesting finding is that drinking can increase the summary scores of the HRQOL-5. One possible explanation for this phenomenon is that Chinese drinking culture was ingrained in the Chinese culture [44]. Some Chinese people think Chinese alcohol could increase pleasure and arousal or decrease pain caused by injury or diseases, which may improve HRQOL and increase summary scores of the HRQOL-5. Of course, due to a range of residual confounding and the design of cross-sectional studies, the observed associations cannot be considered causal associations. Contrary to previous studies [45,46], our results show no statistically significant association between smoking and the summary score of the HRQOL-5. One possible explanation is the limited impact of mentally unhealthy days on the summary score due to its lower factor loading. Some studies found smoking was a predictor only for mental health indicators in healthy days measures [45], and smoking prevalence was higher in participants with poor mental health than those with better mental health [47,48]. Thus, it is essential to use a comprehensive index to evaluate the HRQOL of the population, which can avoid confusion caused by different determinants across different dimensions.

The Healthy China 2030 plan has made population health the ultimate goal of economic development and political reform including justice and equity as one of four core principles [49]. Health equity at the subnational level can be achieved if equipped with a monitoring system for population health status and sufficient data to guide investments. However, designing surveillance instruments must balance collecting needed data with survey costs by including only questions needed to answer research questions [50]. The Chinese version of the HRQOL-5 could substantially decrease participant burden and survey costs by only asking 5 questions. Our study developed a summary index based on the HRQOL-5 to estimate the health status of the population. A multi-data source and nationally representative sample were the greatest strengths of this instrument compared to others, like the SF-36 or EQ-5D. In considering potential intercultural differences, we found the HRQOL-5 could rapidly assess the severity of health states relevant to the Chinese population, and one summary score could capture overall HRQOL via factor analysis, which is important for further development of disease burden research. In addition, the government needs to track changes in health status in different subgroups and identify their determinants due to faster aging and the changing disease spectrum in China. Though Healthy Days measures have been used fairly extensively in various national surveys, previous studies only focused on determinants of a subset of items from the HRQOL-4. If one study found an improvement in one HRQOL-4 item and a decline in another or these HRQOL-4 items were influenced by different determinants, it could be difficult to draw an overall conclusion about the impact on health status. Our results exploratively evaluated risk factors that may influence the summary score of the HRQOL-5 in a Chinese population, which provided a more cohesive picture of HRQOL at the population level. It was particularly useful for setting priorities for health planning to improve primary care in China.

This study had three limitations. First, this study used data from household surveys, which is subject to selection bias because of the exclusion of hospital patients. Second, due to the design of cross-sectional studies, we cannot rule out the potential influence of unmeasured confounders; more studies are needed to draw causal inferences. Finally, since all the participants are from the same city in China, the limited national representation could affect the generalizability of our results. More studies from other Chinese regions are needed to confirm our findings.

In conclusion, this study suggests that it is feasible to summarize 5 HRQOL-5 items in a summary score via factor analysis. The results of the summary score could be used to estimate population-level disability and calculate health-adjusted life expectancy in the future. Our study also showed that age, educational attainments, income, regular exercise, alcohol use, self-reported disease, and self-reported injury were significantly associated with the summary score of the HRQOL-5.

## Supplementary material

10.2196/52019 Multimedia Appendix 1 Supplementary tables.

## Abbreviations

BRFSS: Behavioral Risk Factor Surveillance System
CCDRFS: China Chronic Disease and Risk Factor Surveillance
CDC: Centers for Disease Control and Prevention
CFA: confirmatory factor analysis
CFI: comparative fit index
EFA: exploratory factor analysis
GFI: goodness-of-fit index
HRQOL: health-related quality of life
HRQOL-4: core Health Days measures
HRQOL-5: Chinese version of the core Healthy Days measures
RMSEA: root mean square error of approximation
SF-36: 36-Item Short Form Survey
SRMR: standardized root mean square residual
TLI: Tucker-Lewis index
