# Supplementary material for: Summary Measure of Health-Related Quality of Life and Its Related Factors Based on the Chinese Version of the Core Healthy Days Measures: Cross-Sectional Study
Source: JMIR Public Health Surveill. 2024 Jul 31;10:e52019. doi: 10.2196/52019 (PMC11306403; doi:10.2196/52019)
Supplement: Multimedia Appendix 1 [file publichealth-v10-e52019-s001.docx]

Table S1 The items of the Chinese version of HRQOL-5

| Question | |
| --- | --- |
| Q1: | Would you say that in general your health is excellent, very good, good, fair, or poor?  A. excellent B. very good C. good D. fair E. poor |
| Q2: | Now thinking about your physical health, which includes physical illness and injury, how many days during the past 30 days was your physical health not good?  Number of days ______ |
| Q3: | Now thinking about your mental health, which includes stress, depression, and problems with emotions, how many days during the past 30 days was your mental health not good?  Number of days ______ |
| Q4: | During the past 30 days, approximately how many days did poor physical or mental health keep you from doing your usual activities, such as, work, shopping or recreation?  A. 0 days B. 1-6 days C. 7-14 days D. 15-30 days |
| Q5: | During the past 30 days, approximately how many days did poor physical or mental health keep you from doing your personal care, such as eating, bathing or dressing?  A. 0 days B. 1-6 days C. 7-14 days D. 15-30 days |

Table S2 Results from exploratory factor analysis of HRQOL-5

| Factor | Eigenvalue | Percentage of  explained variance | Accumulated Percentage  of explained variance |
| --- | --- | --- | --- |
| Factor 1 | 2.59 | 51.88 | 51.88 |
| Factor 2 | 0.92 | 18.48 | 70.36 |
| Factor 3 | 0.75 | 15.06 | 85.42 |
| Factor 4 | 0.50 | 10.04 | 95.46 |
| Factor 5 | 0.23 | 4.54 | 100.00 |

Table S3 Multiple linearity diagnosis of factors associated with HRQOL-5

| Variable | VIF |
| --- | --- |
| Gender | 1.55 |
| Age | 1.68 |
| BMI | 1.02 |
| Education | 1.71 |
| Marital Status | 1.10 |
| Average income per capita | 1.45 |
| Residence | 1.13 |
| Physical activity | 1.07 |
| Smoking | 1.38 |
| Drinking | 1.43 |
| Self-reported disease | 1.14 |
| Self-reported injure | 1.01 |
